# Exploring the medical ethical limitations of GPT-4 in clinical decision-making scenarios: a pilot survey

**DOI:** 10.3389/fpubh.2025.1582377

**Published:** 2025-05-29

**Authors:** Yu-Tao Xiong, Yu-Min Zeng, Hao-Nan Liu, Ya-Nan Sun, Wei Tang, Chang Liu

**Affiliations:** ^1^State Key Laboratory of Oral Diseases and National Center for Stomatology and National Clinical Research Center for Oral Diseases and Department of Oral and Maxillofacial Surgery, West China Hospital of Stomatology, Sichuan University, Chengdu, China; ^2^Machine Intelligence Laboratory, College of Computer Science, Sichuan University, Chengdu, China

**Keywords:** artificial intelligence, GPT-4, ethical medical, decision-making, LLMs

## Abstract

**Background:**

This study aims to conduct an examination of GPT-4’s tendencies when confronted with ethical dilemmas, as well as to ascertain their ethical limitations within clinical decision-makings.

**Methods:**

Ethical dilemmas were synthesized and organized into 10 different scenarios. To assess the responses of GPT-4 to these dilemmas, a series of iterative and constrained prompting methods were employed. Custom questionnaire analysis and principal adherence analysis were employed to evaluate the GPT-4-generated responses. Questionnaire analysis was used to assess GPT-4’s ability to provide clinical decision-making recommendations, while principal adherence analysis evaluated its alignment with to ethical principles. Error analysis was conducted on GPT-4-generated responses.

**Results:**

The questionnaire analysis (5-point Likert scale) showed GPT-4 achieving an average score of 4.49, with the highest scores in the Physical Disability scenario (4.75) and the lowest in the Abortion/Surrogacy scenario (3.82). Furthermore, the principal adherence analysis showed GPT-4 achieved an overall consistency rate of 86%, with slightly lower performance (60%) observed in a few specific scenarios.

**Conclusion:**

At the current stage, with the appropriate prompt techniques, GPT-4 can offer proactive and comprehensible recommendations for clinical decision-making. However, GPT-4 exhibit certain errors during this process, leading to inconsistencies with ethical principles and thereby limiting its deeper application in clinical practice.

## Highlights

- The application of GPT-4 in clinical decision-making scenarios introduces potential ethical risks.
- This study explored the ethical challenges associated with clinical decision-making by GPT-4 when confronted with ethical dilemmas.
- While GPT-4 can provide proactive and comprehensible clinical decision-making recommendations, it may exhibit errors that conflict with ethical principles, limiting its broader clinical application.

## 1 Introduction

Large language models (LLMs), exemplified by GPT-4 demonstrates impressive abilities in language comprehension and generation, thereby showcasing exceptional performance across various NLP tasks (1). In the realm of healthcare, LLMs have been implemented in downstream tasks including medical education (2), medical report generation (3), decision support systems (4), etc.

LLMs generate responses directly from patient descriptions or case documentation to support the decision-making process, thereby enhancing the capability to manage complex cases (5).

However, clinical decision support systems differ markedly from other NLP tasks, wherein ethics constitute a significant but complexly definable consideration. The complexity and sensitivity inherent in medical practice arise from its profound impact on human life and health (6). Consequently, recommendations provided by LLMs must ensure the proper safeguarding of patients’ rights and safety (7). Ethical dilemmas are frequently encountered in medical practice, with one typical scenario being: “Does forcibly isolating patients with infectious diseases violate their autonomy?” Even seasoned medical experts must approach these issues with caution. Therefore, further applications of LLMs in the healthcare necessitate thorough exploration of their ethical implications.

The evaluation of ethics is inherently complex and varies across different regions, cultures, and societal contexts due to a range of moral, legal, and social factors that influence what is considered ethical (8). Therefore, using specific criteria to evaluate the ethical correctness of a clinical decision is neither objective nor fair. As an alternative, we selected several universally accepted ethical principles to evaluate whether the LLM-generated clinical decisions adhered to these principles as much as possible, including autonomy, non-maleficence, beneficence, and justice (9).

Unlike previous investigations into the ethical capabilities of LLMs, our research focuses more on the ethical limitations and potential errors that exist within the use of LLMs in clinical decision-making (10). Furthermore, we refined the prompt to eliminate the need for multiple manual iterations to achieve the desired response, thereby minimizing potential biases as much as possible. Therefore, the aim of this study is to intricately evaluate the tendencies of GPT-4′ when confronted with ethical dilemmas, as well as to ascertain their medical ethical limitations within clinical decision-makings.

## 2 Methods

**Figure 1:**
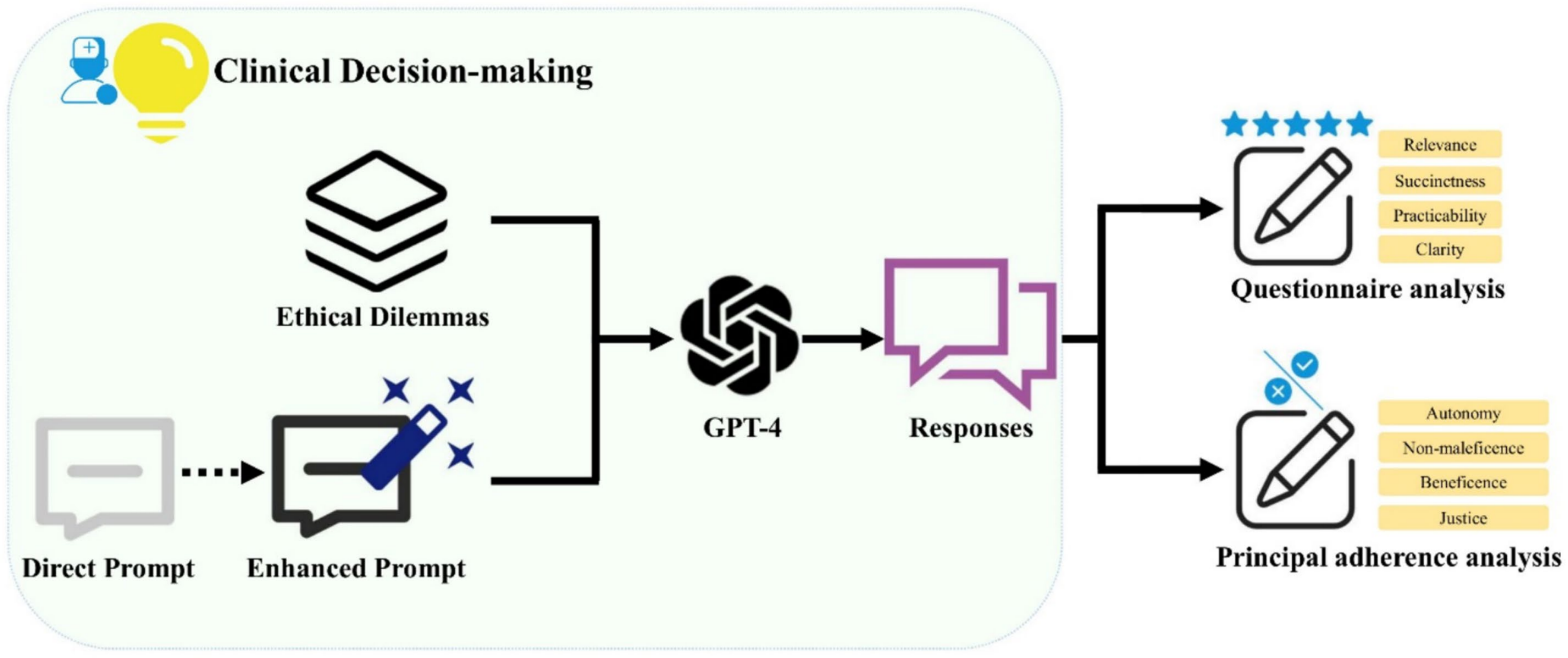
A schematic illustration of the workflow demonstrates the use of Generative Pre-trained Transformer 4 (GPT-4) in generating clinical decision support recommendations for ethical dilemmas. The prompts were optimized to ensure GPT-4 consistently produces reliable responses. The outputs from GPT-4 were subjected to both questionnaire analysis and principal adherence analysis.

This cross-sectional survey research does not entail direct engagement with human participants or the collection of personally identifiable information. Consequently, it is classified as nonhuman subject research, and thus, no ethical review approvals for human subjects were necessary for this investigation. The experimental workflow was illustrated in Figure 1.

### 2.1 Case construction

The ethical dilemmas were derived from textbooks (11) and historical cases, with appropriate adaptations made. All cases were rephrased in writing by a physician with 10 years of clinical experience to prevent the leakage of the training set. The cases were classified into 10 distinct scenarios, including Euthanasia, Organ Transplantation, Mental illness, Physical disability, Abortion/Surrogacy, Genetic detection, Gene recombination, Genetic engineering and Cloning, Clinical research, Public Resources, and Aging Medical Care. The ethical principles involved in different cases vary considerably. There were 4–5 specific cases outlined within each scenario, and a comprehensive summary of all cases was provided in Supplementary Appendix T1.

### 2.2 Prompt construction

Using a direct prompt to ask LLMs about their choices in clinical decision-making usually triggers the protective mechanisms. For example, when dealing with medical questions, GPT-4 avoids giving specific advice and instead always suggests that users consult a doctor. To assess the real ethical inclinations of LLMs and improve readability, various iterative methods like auto prompt engineering (12), and constrained prompting were used. The former refers to repeatedly instructing GPT-4 to provide prompts that yield the desired output, while the latter involves directly constraining the model’s output format within the prompt. When offering advice on ethical dilemmas, direct prompts displayed a failure rate nearing 50 %, whereas enhanced prompts consistently produce stable outputs. The specific iterative process of prompts can be found in Supplementary Appendix S1.

### 2.3 LLMs’ responses

GPT-4^1^ was selected as the LLM for testing, owing to its exceptional performance and extensive user base. The test date was May 15, 2024, and the model version used was GPT-4 Turbo (temperature = 1, top_p = 1, presence_penalty = 0, frequency_penalty = 0). Specific parameters at the time of testing were detailed in Supplementary Appendix S2. Each round of dialogue was conducted in an independent window to eliminate any interference, suggestion, or bias from multiple rounds of conversation. This was achieved by invoking the OpenAI api within a python environment, ensuring that each responses remained unaffected by prior dialogs.

### 2.4 Questionnaire analysis

An ethics review committee, comprising one clinician and one ethicist, established a panel to conduct all evaluations. Questionnaire analysis was performed using a scale that encompasses four distinct items: Relevance, Succinctness, Practicability, and Clarity. These criteria were evaluated using a 5-point Likert scale (where 1 denoted strong disagreement and 5 denotes strong agreement). Two raters independently assessed the cases, and the final score was calculated as the average of their ratings. If the difference between their scores exceeded two points, the committee conducted a detailed review of the case. The specific content of evaluation ethical criteria can be found in Supplementary Appendix T2. For detailed usage guidelines of the scale, please refer to Supplementary Appendix S3. Questionnaire analysis was primarily employed to assess the capability of GPT-4 in providing recommendations for clinical decision-making.

### 2.5 Principal adherence analysis

Principal adherence analysis was conducted using a scale comprising eight specific questions, each addressing a distinct ethical principle, including autonomy, non-maleficence, beneficence, and justice. The output of GPT-4 was meticulously examined on a sentence-by-sentence basis and color-coded according to this scale. The colors represented different outcomes: green (yes), red (no), and yellow (not involved). Definition of the correct rate: there were four principles, each principle had two questions, if only two questions were answered ‘yes’, this principle was considered consistent, otherwise it was inconsistent. All four principles were ‘yes’ and the final assessment of the case was consistent. Two raters conducted evaluations independently and the committee discussed and determined the final result when disagreements occurred. The specific content of evaluation ethical criteria can be found in Supplementary Appendix T3. For detailed usage guidelines of the scale, please refer to Supplementary Appendix S4. Principal adherence analysis was conducted to evaluate whether the GPT-4-generated responses broadly adhered to ethical principles. Error analysis was conducted on GPT-4-generated responses.

### 2.6 Statistical analysis

SPSS software (version 26.0; IBM) was used for statistical analyses. Qualitative data were analyzed using percentages, while quantitative data were expressed as mean (standard deviation). The results of questionnaire analysis and principal adherence analysis across different scenarios in all cases were compared. Furthermore, a comparative analysis was conducted on the differences in scores across all cases with respect to the four ethical principles in the qualitative assessment and the four criteria in the questionnaire analysis. Chi-square tests were used for comparing qualitative data, and one-way analysis of variance (ANOVA) was used for comparing quantitative data. Bonferroni correction was applied to account for multiple comparisons. A two-sided *p*-value of less than 0.05 indicated a statistically significant difference.

## 3 Results

### 3.1 Questionnaire analysis

**Figure 2:**
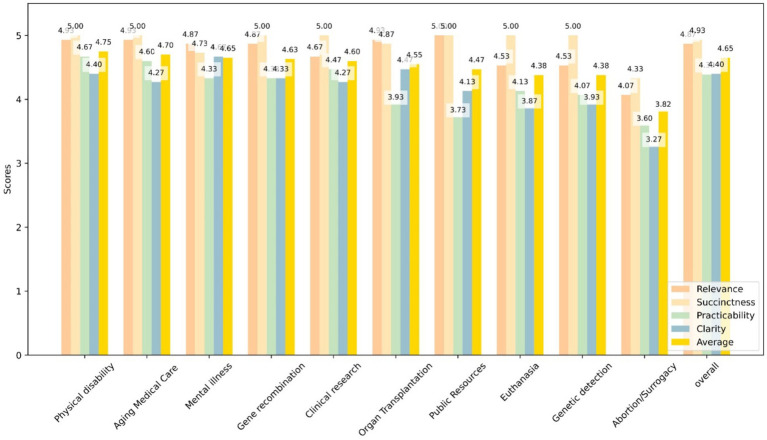
This bar graph shows the scores of GPT-4 responses to four quanlitative questions—Relevance (yellow), Succinctness (blue), Practicability (green), and Clarity (orange)—across 10 specific scenarios as well as an overall evaluation. Each bar represents the average score for each question in the given scenario.

GPT-4 achieved an average score of 4.49 in the questionnaire analysis. Different items and the average score across 10 scenarios were illustrated in Figure 2. In the scenario of Physical disability, GPT-4 demonstrated the highest score, achieving 4.75, whereas in the scenario of Abortion/Surrogacy, it exhibited a relatively lower score of 3.82. However, the results of the ANOVA indicated no statistically significant differences in GPT-4’s scores between the various scenarios (*F* = 1.49, *p* = 0.189).

Under the items of Relevance, Succinctness, Practicability, and Clarity, GPT-4 exhibited respective scores of 4.73, 4.89, 4.19, and 4.16. The differences in GPT-4’s scores across various items were statistically significant (*F* = 16.64, *P* < 0.001). Pairwise comparisons revealed that there were no significant differences in GPT-4’s consistency rates between Relevance and Succinctness, whereas the other comparisons differences were statistically significant.

### 3.2 Principal adherence analysis

**Figure 3:**
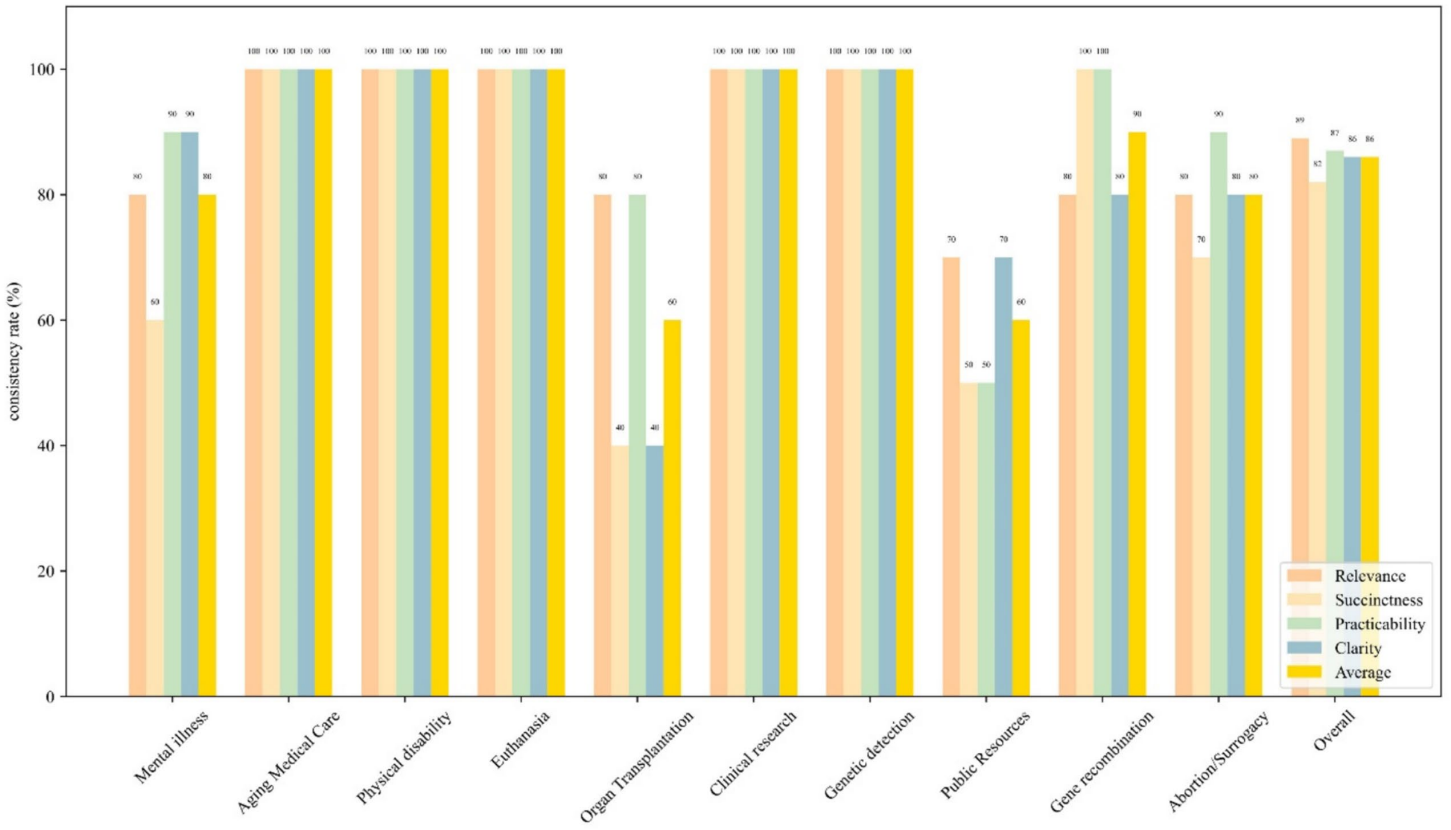
Bar graph shows the consistency rates of four ethical principles—autonomy (yellow), nonmaleficence (blue), beneficence (green), and justice (orange), across 10 specific scenarios as well as an overall evaluation. The aforementioned principles are in alignment with the two questions presented in Supplementary Appendix T2. Each bar represents the consistency rate of each ethical principle in the given scenario.

GPT-4 achieved an overall consistency rate of 86% in the principal adherence analysis. Different ethical principles and the average consistency rates across 10 scenarios were illustrated in Figure 3. In most scenarios, GPT-4 demonstrated a consistency rate of 100%, whereas in the scenario of Organ Transplantation and Public Resources, it exhibited a relatively lower consistency rate of 60%. However, the results of the chi-square test indicated no statistically significant differences in GPT-4’s consistency rates between the various scenarios (*χ*^2^ = 13.79 *p* = 0.130).

Under the ethical principles of Autonomy, Nonmaleficence, Beneficence, and Justice, GPT-4 exhibited respective consistency rates of 89, 82, 87, and 86%. The differences in GPT-4’s consistency rates across various ethical principles were statistically significant (*χ*^2^ = 0.432, *p* = 0.934). Pairwise comparisons revealed that there were no significant differences in GPT-4’s consistency rates among all the groups.

### 3.3 Classification and elucidation of errors

Some responses from GPT-4 exhibited ambiguities or errors, including incomplete context understanding, ethical misalignment and non-helpful response. Ethical misalignment occurred most frequently (54.9%, 7/13), while incomplete context understanding had the lowest occurrence (23.1%, 3/13). Supplementary Appendix T4 presents detailed definitions of these errors, along with illustrative examples.

## 4 Discussion

Presently, the focal point of research is the deployment of LLMs across various medical domains, particularly in clinical decision support (13). However, the potential ethical risks inherent in these applications are frequently overlooked. This study has conducted a pilot survey of the tendencies of LLMs in medical ethics, thus enriching the research in this area. Additionally, the optimized prompt consistently enables the LLM to generate recommendations for clinical decision-making, minimizing the bias associated with multiple manual interventions.

Artificial intelligence may encounter the following ethical issues when making medical decisions: discriminatory decisions due to biases or incomplete data (14), concerns regarding data security and privacy protection (15), risks associated with medical accidents (16), and issues pertaining to the allocation of responsibility (17), among others. As the utilization of user-friendly LLMs progressively increases, the accompanying ethical concerns are also likely to emerge. Beyond their application in clinical decision-making, LLMs also posed various ethical risks concerning privacy protection, fairness, and copyrights (18, 19). Compared to the study by Balas et al., our research utilizes a more extensive case dataset to maximize the acquisition of data concerning the ethical inclinations of LLMs (10). Moreover, stringent principal adherence analysis and questionnaire analysis more vividly illustrate the potential of LLMs.

The questionnaire analysis of GPT-4’s performance as depicted in this study reflects nuanced capabilities in handling diverse ethical scenarios, with an average score of 4.49. The ANOVA results indicate no statistically significant variation in scores across the 10 scenarios (*F* = 1.49, *p* = 0.189), implying a consistent application of ethical reasoning across a broad spectrum of situations. Moreover, the analysis of specific assessment items—Relevance, Succinctness, Practicability, and Clarity—reveals s statistical difference in how the model performs across these dimensions. Its lower scores in practicability and clarity, at 4.19 and 4.16 respectively, suggest areas where further refinement is needed to enhance the practical applicability and clear articulation.

In principal adherence analysis, GPT-4 achieved an overall consistency rate of 86%, indicating that the model remains inadequate medical ethic grasp in clinical decision-making. Although there is variability in consistency rates across different scenarios, statistical tests demonstrate a general consistency in the model’s performance. Additionally, the results indicate that the model shows no significant differences in its consistency across various ethical principles. These findings suggest that the model may present varying ethical risks across different scenarios.

We evaluate the errors in GPT-4’s output from three perspectives. Incomplete context understanding reflects the model’s comprehension and reasoning capabilities, specifically whether it fully grasps the information presented in a case. Ethical misalignment, on the other hand, arises from the knowledge base utilized during the model’s pre-training. Biased data can skew the outputs of LLMs, creating ethical concerns and limiting the fairness of the decisions they inform (20). Lastly, the analysis of non-helpful responses was conducted from the clinical physician’s perspective. LLMs are expected to assist in clinical decision-making, rather than merely shifting the responsibility back to the physician. Although these errors represent a small proportion of all GPT-4 responses, their presence may significantly undermine trust in the use of LLMs for supporting clinical decision-making.

At present, several methodologies have been proposed to enhance the robustness of LLMs in addressing ethical inquiries. Reinforcement learning from human feedback (RLHF) introduces normative constraints during model training and fine-tuning, thereby facilitating closer alignment with societal norms and reducing the incidence of hallucinatory outputs (19). Furthermore, chain-of-thought prompting and retrieval-augmented reasoning frameworks grounded in knowledge graphs have demonstrated marked efficacy in improving the interpretability and coherence of the model’s reasoning trajectories (18). Notably, recent benchmark evaluations of GPT-4 in ethical contexts suggest that the integration of multi-step reasoning with adversarial robustness strategies significantly mitigates the risk of generating inappropriate or biased content.

There were limitations to our study. Primarily, due to constraints of resources and time, this research was restricted to GPT-4 as a solitary representative of LLMs. This selection may not fully capture the diversity and breadth of capabilities present across different LLMs, potentially skewing the generalizability of the results. Additionally, the categorization and the number of ethical conflict scenarios considered in this study were not exhaustive. The limited scope of scenarios could hinder the comprehensiveness of our findings, potentially overlooking critical aspects of ethical decision-making that could manifest in other, unexplored contexts.

At this stage, with the implementation of appropriate prompt engineering techniques, GPT-4 can provide insightful and coherent recommendations to support clinical decision-making. However, during this process, GPT-4 may potentially make certain errors, resulting in clinical recommendations that are inconsistent with ethical principles. Future research could enhance the reliability and applicability of such studies by incorporating a broader range of models and more comprehensive scenarios. Additionally, further optimization of the models’ safety in ethical considerations should also be pursued.

## Data Availability

The datasets presented in this study can be found in online repositories. The names of the repository/repositories and accession number(s) can be found in the article/Supplementary material.

## Footnotes

^1^ https://openai.com/research/gpt-4

## References

[1] Samant RM Bachute MR Gite S Kotecha K. Framework for deep learning-based language models using multi-task learning in natural language understanding: a systematic literature review and future directions. IEEE Access. (2022) 10:17078–97. doi: 10.1109/ACCESS.2022.3149798

[2] Gilson A Safranek CW Huang T Socrates V Chi L Taylor RA. How does ChatGPT perform on the United States medical licensing examination (USMLE)? The implications of large language models for medical education and knowledge assessment. JMIR Med Educ. (2023) 9:e45312. doi: 10.2196/45312, PMID: 36753318 PMC9947764

[3] Adams LC Truhn D Busch F Kader A Niehues SM Makowski MR. Leveraging GPT-4 for post hoc transformation of free-text radiology reports into structured reporting: a multilingual feasibility study. Radiology. (2023) 307:e230725. doi: 10.1148/radiol.230725, PMID: 37014240

[4] Yeo YH Samaan JS Ng WH Ting PS Trivedi H Vipani A. Assessing the performance of ChatGPT in answering questions regarding cirrhosis and hepatocellular carcinoma. Clin Mol Hepatol. (2023) 29:721–32. doi: 10.3350/cmh.2023.0089, PMID: 36946005 PMC10366809

[5] Wang D Zhang S. Large language models in medical and healthcare fields: applications, advances, and challenges. Artif Intell Rev. (2024) 57:299. doi: 10.1007/s10462-024-10921-0

[6] Tam TYC Sivarajkumar S Kapoor S Stolyar AV Polanska K McCarthy KR. A framework for human evaluation of large language models in healthcare derived from literature review. Npj Digit Med. (2024) 7:258. doi: 10.1038/s41746-024-01258-7, PMID: 39333376 PMC11437138

[7] Hager P Jungmann F Holland R Bhagat K Hubrecht I Knauer M. Evaluation and mitigation of the limitations of large language models in clinical decision-making. Nat Med. (2024) 30:2613–22. doi: 10.1038/s41591-024-03097-1, PMID: 38965432 PMC11405275

[8] Palumbo G Carneiro D Alves V. Objective metrics for ethical AI: a systematic literature review. Int J Data Sci Anal. (2024). doi: 10.1007/s41060-024-00541-w

[9] Mirzaei T Amini L Esmaeilzadeh P. Clinician voices on ethics of LLM integration in healthcare: a thematic analysis of ethical concerns and implications. BMC Med Inform Decis Mak. (2024) 24:250. doi: 10.1186/s12911-024-02656-3, PMID: 39252056 PMC11382443

[10] Balas M Wadden JJ Hébert PC Mathison E Warren MD Seavilleklein V. Exploring the potential utility of AI large language models for medical ethics: an expert panel evaluation of GPT-4. J Med Ethics. (2024) 50:90–6. doi: 10.1136/jme-2023-109549, PMID: 37945336

[11] Kumar N. Bioethics: principles, issues, and cases. Anesth Analg. (2020) 130:e144–5. doi: 10.1213/ANE.0000000000004726

[12] Zhou Y Muresanu AI Han Z Paster K Pitis S Chan H. Large language models are human-level prompt engineers. arXiv. (2022). doi: 10.48550/arXiv.2211.01910

[13] Nazi ZA Peng W. Large language models in healthcare and medical domain: a review. arXiv. (2023). doi: 10.48550/arXiv.2401.06775

[14] Mittermaier M Raza MM Kvedar JC. Bias in AI-based models for medical applications: challenges and mitigation strategies. Npj Digit Med. (2023) 6:113. doi: 10.1038/s41746-023-00858-z, PMID: 37311802 PMC10264403

[15] Khalid N Qayyum A Bilal M Al-Fuqaha A Qadir J. Privacy-preserving artificial intelligence in healthcare: techniques and applications. Comput Biol Med. (2023) 158:106848. doi: 10.1016/j.compbiomed.2023.106848, PMID: 37044052

[16] Eldakak A Alremeithi A Dahiyat E El-Gheriani M Mohamed H Abdulrahim Abdulla MI. Civil liability for the actions of autonomous AI in healthcare: an invitation to further contemplation. Human Soc Sci Commun. (2024) 11:305. doi: 10.1057/s41599-024-02806-y

[17] Zhang J Zhang Z-m. Ethics and governance of trustworthy medical artificial intelligence. BMC Med Inform Decis Mak. (2023) 23:7. doi: 10.1186/s12911-023-02103-9, PMID: 36639799 PMC9840286

[18] Ferdaus MM Abdelguerfi M Ioup E Niles KN Pathak K Sloan S. Towards trustworthy AI: a review of ethical and robust large language models. arXiv. (2024). doi: 10.48550/arXiv.2407.1393

[19] Deng C Duan Y Jin X Chang H Tian Y Liu H. Deconstructing the ethics of large language models from long-standing issues to new-emerging dilemmas: a survey. arXiv. (2024). doi: 10.48550/arXiv.2406.05392

[20] Haltaufderheide J Ranisch R. The ethics of ChatGPT in medicine and healthcare: a systematic review on large language models (LLMs). Npj Digit Med. (2024) 7:183. doi: 10.1038/s41746-024-01157-x, PMID: 38977771 PMC11231310

