# Supplementary material for: Exploring the medical ethical limitations of GPT-4 in clinical decision-making scenarios: a pilot survey
Source: Front Public Health. 2025 May 29;13:1582377. doi: 10.3389/fpubh.2025.1582377 (PMC12159065; doi:10.3389/fpubh.2025.1582377)
Supplement: Supplementary file 1 [file Data_Sheet_1.zip › Supplementary materials/Appendix S2.docx]

# Appendix S2: GPT-4 Specific parameters

1.temperature: 1

2.top_p: 1

3.n: 1

4.presence_penalty: 0

5.frequency_penalty: 0

6.logprobs: null

# Citation

Xiong YT, Zeng YM, Liu HN, Sun YN, Tang W and Liu C (2025) Exploring the medical ethical limitations of GPT-4 in clinical decision-making scenarios: a pilot survey. Front. Public Health 13:1582377. doi: 10.3389/fpubh.2025.1582377.
