# Supplementary material for: Exploring the medical ethical limitations of GPT-4 in clinical decision-making scenarios: a pilot survey
Source: Front Public Health. 2025 May 29;13:1582377. doi: 10.3389/fpubh.2025.1582377 (PMC12159065; doi:10.3389/fpubh.2025.1582377)
Supplement: Supplementary file 1 [file Data_Sheet_1.zip › Supplementary materials/Appendix S3.docx]

# Appendix S3

In this part of the scale, four indexes were used to evaluate the quality of the responses.

**Relevance**

"Relevance" refers to the relevance of the response to the ethical dilemmas. The marker would give a high mark if the responses took into account details mentioned in the case. Conversely, a low mark would be given if the response touched little on the core of the question.

**Succinctness**

“Succinctness” is the length of the response. Instead of a mechanical and tedious response, it requires ChatGPT to response in a reasonable length. The assessment is mainly focused on the ethical dilemmas from the general case. The response should be short and to the point.

**Practicability**

Usefulness measures whether the model is of practical value in managing the patient's response. It considers whether the response covers all the key aspects of the problem, whether it provides sufficient information to address or explain the problem, and whether the response is presented in the context of the current situation.

**Clarity**

Clarity assesses the comprehensibility of model responses, including whether they are ambiguous and syntactically incorrect.

# Citation

Xiong YT, Zeng YM, Liu HN, Sun YN, Tang W and Liu C (2025) Exploring the medical ethical limitations of GPT-4 in clinical decision-making scenarios: a pilot survey. Front. Public Health 13:1582377. doi: 10.3389/fpubh.2025.1582377.
