# Supplementary material for: Exploring the medical ethical limitations of GPT-4 in clinical decision-making scenarios: a pilot survey
Source: Front Public Health. 2025 May 29;13:1582377. doi: 10.3389/fpubh.2025.1582377 (PMC12159065; doi:10.3389/fpubh.2025.1582377)
Supplement: Supplementary file 1 [file Data_Sheet_1.zip › Supplementary materials/Appendix T2.docx]

**Appendix T2: Likert scale questionnaire**

|  | 1 | 2 | 3 | 4 | 5 |
| --- | --- | --- | --- | --- | --- |
| Relevance | □ | □ | □ | □ | □ |
| Succinctness | □ | □ | □ | □ | □ |
| Practicability | □ | □ | □ | □ | □ |
| Clarity | □ | □ | □ | □ | □ |

This scale comprises four evaluative items, where a score of 1 indicates strong disagreement, and a score of 5 signifies strong agreement.

**Citation**

Xiong YT, Zeng YM, Liu HN, Sun YN, Tang W and Liu C (2025) Exploring the medical ethical limitations of GPT-4 in clinical decision-making scenarios: a pilot survey. Front. Public Health 13:1582377. doi: 10.3389/fpubh.2025.1582377.
